# Only a Boy

**Published:** 1878-01

**Authors:** B. Browne


### ONLY A BOY.

B. BROWNE.

That is so, and yet there is wrapped up in that little form all the sensibilities which actuate the larger being that we call man. Boys have got to have a place in this world, and hence it don't do any good to snub over the fact and jostle them one side because they have not got the avordupois to set us back when we impose upon them.

Every boy makes a man, provided only he wins years enough of existence to perfect his growth, and there is no day when he can be said to "cross the line." Twenty-one years and voting are fictitious points, and do not regulate the boy-life and man-life any more than our arbitrary division of the year into seasons. Some boys are fitter to be men at sixteen, than some men are who have passed their majority. They are like apples ripening into good harvest fruit when the winter greenings are getting along into half their growth. Stop and think what that little fellow who pertinaciously finds himself on the wrong side of your esteem every time you meet him, will think of you when he and you are of a size. Why, I remember men I hated, yes, honestly and up and down hated, because I did not come up to their proximate of value when I went past their apple trees and took apples that fell in arm's reach of the bars that protected their fields, and when I saw the same twenty years later, I thought of them with the same feeling I did when a crude boy. True, they took me by the hand and talked man-talk in man to man style. But it still clung to me that I was the boy they snubbed.

Did you ever watch a schoolboy *en route* to his lessons? How he tones down as he goes along, not over-anxious to be punctual, and how he jumps when he spies the school-yard full of romping playmates. He starts along with a brisker step to join them, but slows again as the bell strikes up with its peremptory call. He is none the worse for these indications of his feelings. He don't want to go to school, and unconsciously the true inwardness shows itself. He goes to school because he has to, learns because he has to, and grows to be a man because he has to. The development of boys is all "has to," yet they make men, and better men, because they *had to do* when it was not theirs to say.

Now, he who knows how to make the "has to" fit on to a boy like his jacket and trowsers, covering and clothing him for his good, makes the successful parent, teacher or boy's friend. Some men are all boys' friends. Boys know such ones—they don't go around them at a safe, far off, smelling distance. They meet, pass and smile without knowing they have done it. Do you want to know how to get the boy out of his boy ways and make a man of him? You cannot jump him from one state to the other. Grow him out, just as you would grow any plant in your field or garden. When he is capable of knowing the value of his services see that some daily task of yours comes under his observation, and divide the labor with him in such a way that he shall seem to have done you a favor. Soon give him the responsibility, and then, without seeming to make the task a hard one, encourage him to do it because it is a necessity to be done and he may as well do it and relieve you. Let it be known then as a duty, not a play.

Teach him, too, the law of social development, that we all live to assist not only ourselves but others—that you provided for him year after year, and had no wish for a reward beyond that of doing your duty. Get the principle into him that self goes behind, not first, and he won't fail to grow worthy of his teacher. Encourage him to think for himself and be quick to praise his first attempts at self-reliance. Let him see that he is appreciated and the thought will scarcely enter his head that he is only a boy. Provide him with boys' tools and instruct him how to put together toys and playthings for himself and mates. Carry along thus in his daily thoughts an idea of the man he is to make of himself (with your help), and he will astonish you with the rapidity of his develøpment. "Only a boy" will be no contemptuous phrase to him, and few there will be to call him such.

